# Se Nanowire Crystal Formation via Oxidation of 2D HfSe_2_: A Solid-State, In Situ Reaction Coupling for Heterogeneous Integration Technologies

**DOI:** 10.1021/acsanm.5c00308

**Published:** 2025-04-09

**Authors:** Sunvir Sahota, Irina Chircă, Oliver J. Burton, Hao Yu, Max Rimmer, Jinfeng Yang, Kyungseo Park, Arthur Summers, Siddika Mertdinc-Ulkuseven, Matthew Lindley, Sarah J. Haigh, Stephan Hofmann

**Affiliations:** † Department of Engineering, University of Cambridge, Cambridge CB3 0FA, U.K.; ‡ Department of Materials, University of Manchester, Oxford Road, Manchester M13 9PL, U.K.; § Metallurgical and Materials Engineering Department, Istanbul Technical University, Maslak, Istanbul 34469, Turkey

**Keywords:** 2D materials, 1D materials, nanowires, HfSe_2_, oxidation, trigonal Se, crystal growth

## Abstract

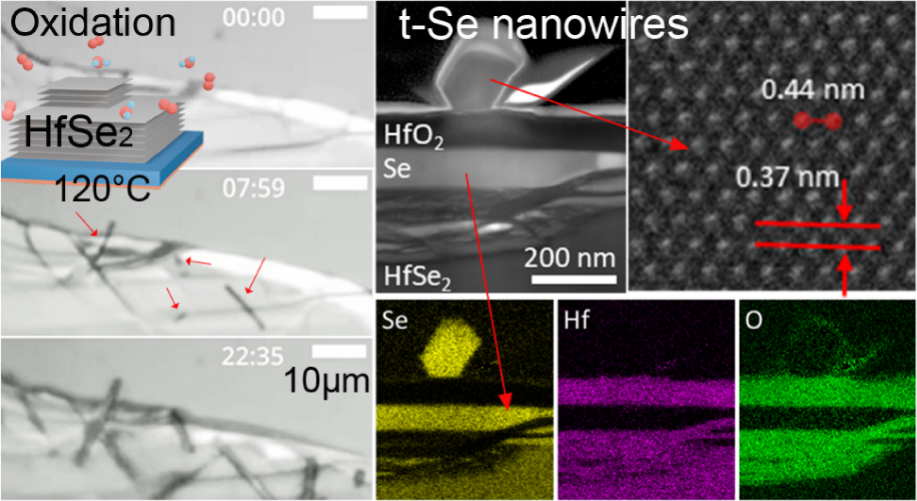

Effective heterogeneous integration of low-dimensional nanomaterials in applications ranging from quantum electronics to biomedical devices requires a detailed understanding of different formation and interfacing reactions and the ability to synergize these processes. We report the formation of 1D Se nanowires via low-temperature (30–150 °C) atmospheric oxidation of 2D HfSe_2_ crystals. The localized, surface-bound process starting from exfoliated HfSe_2_ flakes on a SiO_2_/Si wafer support does not involve wet chemistry and allows us to implement optical operando reaction screening and explore the relevant parameter space and underpinning mechanisms. Hf oxidation frees Se at the buried hafnia–HfSe_2_ interface, which segregates as amorphous Se, forming aggregates, blisters, and interfacial films. We show that upon diffusion to the stack surface, this Se can crystallize into trigonal Se nanowires with diameters ranging from ∼45 nm to 1.9 μm and lengths up to 43 μm depending on temperature and process time. We discuss the coupled reaction kinetics and pathways for application-relevant integrated process designs and connect diverse literature on the oxidation of transition metal dichalcogenides, Se polymerization and crystallization studies, and prior synthetic strategies for producing Se nanowires.

## Introduction

A key challenge to harnessing the unique properties of one-dimensional (1D) and two-dimensional (2D) materials is achieving heterogeneous integration for next-generation, scalable device technologies.^1−3^ This requires a detailed understanding of the underpinning reaction mechanisms governing material formation, conversion, and interfacial interactions. Given the crystal anisotropy, reduced dimensionality, exposed surface character, and many known extrinsic dependencies of such quantum materials, these reaction mechanisms can be as unique as the material properties but remain much less explored. The fundamental reaction of material oxidation exemplifies this, with many new phenomena discovered as research on material stability has shifted from 3D to 2D, down to the monolayer level.^4−6^ For layered transition metal dichalcogenides (TMDs),^7^ oxidation studies have also been technologically driven, from the need for dielectric interfacing and doping,^8,9^ to device stacks for neuromorphic computing.^10−12^ TMDs like HfS_2_ and HfSe_2_ have been investigated due to their potential of forming a stable, high-κ native oxide, in this case hafnia.^13−15^

The growth of such a native metal oxide layer, however, requires the extraction of the chalcogen species from the layer stack. Thermal annealing and other oxidative exposures are often found to lead to a “blistering” or roughening effect of the stack surface, suggesting a displacement of the chalcogen followed by nonuniform trapping and clustering.^16−18^ Depending on the oxidation parameters and reaction kinetics, for HfS_2_ and other sulfides, sulfur can volatilize as gaseous SO_2_, but the result can also be liquid or solid SO_3_ or a solid oxy-sulfide. In contrast, for selenides like HfSe_2_ and TiSe_2_, the segregation of less volatile Se into amorphous Se (a-Se) layers or aggregates has been reported, accumulating either in-between layers or as particles on the stack surface.^16,17,19^ Such Se segregation is typically undesirable,^20^ yet its thermal activation, direct link to oxidation, and straightforward detection can open different functionalities such as temperature sensing.^17^

Here, we demonstrate that Se segregation during the low-temperature atmospheric oxidation of exfoliated HfSe_2_ flakes can lead to the formation of crystalline trigonal Se (t-Se) nanowires (NWs). Se is among the most important semiconductor materials, particularly due to its high photoconductivity and use in photocopying and imaging systems.^21^ Se is also a model material due to its divalency and the resulting unique polymeric structure, with the monomer being a single Se atom, [Se]_*n*_, and the thermodynamically most stable trigonal crystal phase (t-Se) consisting of helically arranged atomic chains.^22,23^ Accordingly, there have been many Se polymerization and crystallization studies,^24−26^ also reporting high aspect ratio t-Se needle and tubular crystal formation due to the structural anisotropy giving rise to strong growth anisotropy. Various synthetic strategies have been reported for t-Se NWs, motivated by structural control at the nanoscale and thus property tailoring with potential applications ranging from sensor arrays, photo/electro-catalysis, biomedical devices, data storage, and piezoelectronics to integrated optoelectronics.^27−32^ The emphasis to date has been on bulk production, and the techniques involved make direct access to the underpinning crystal growth kinetics difficult. Hence, the understanding particularly of the NW formation remains incomplete. Common to most approaches is a source of a-Se, whether from chemical solution^27^ or vapor phase,^33−35^ followed by nucleation of t-Se and crystalline NW formation. In our approach, a-Se is a reaction byproduct of HfSe_2_ oxidation, with the segregation or condensation reaction drawing parallels to solid-phase epitaxy.^36^ By controlling the reaction conditions of HfSe_2_ oxidation, we can drive localized growth of t-Se NWs. Our synthesis approach is interesting not only for future integrated device processing but also as a model system to achieve direct experimental access to SeNW formation mechanisms and thus to foster a deeper understanding of the process. We implement optical operando reaction screening combined with complementary postprocess electron microscopy (TEM and SEM), scanning probe (AFM), and Raman characterization to explore the relevant parameter space and connect the diverse existing literature findings.

## Experimental Section

Sample preparation: HfSe_2_ crystals (HQ graphene) were micromechanically exfoliated (Ultron Systems adhesive tape) and stored in an inert Ar atmosphere inside a glovebox to suppress premature oxidation during extended ambient exposure.^16^ Air exposure during sample transfer was limited to less than 60 min, which was found not to significantly influence the key findings. The SiO_2_ (285 nm)/Si wafer substrates were cleaned with acetone and IPA and then plasma treated (oyxgen, 100 W).

A custom optical setup, consisting of a standard microscope fitted with a liquid crystal tunable filter and a monochrome camera (Thor laboratories Kurios, 420–730 nm), and a Linkam heating stage were used for operando observation of the atmospheric HfSe_2_ oxidation. The implemented process was 50 °C/min ramp, held at anneal temperature, and left to cool in ambient. We operated in a bright-field reflectance mode, illuminated with a Thorlabs SLS201/M in the 420–730 nm range with an approximate total output power of 10 mW. Sequential optical losses in the system reduced this to an upper bound of approximately 1 mW distributed over a 1 × 1 mm area on the sample.

The samples were characterized by AFM (MFP-3D Asylum System and Bruker Icon), SEM (Zeiss Gemini 300 SEM), and Raman spectroscopy (Renishaw inVia, 100× objective, at 514, 532, or 633 nm excitation, and a typical approximate power density of 0.06 mW cm^–2^). Cross-sectional FIB lamellae were fabricated using a FEI Helios 660 dual-beam FIB. Prior to FIB processing, the samples were coated with carbon via thermal deposition and Pt over the target area by the FIB to protect the surface from ion beam damage. Bulk etching and thinning were performed at 30 kV accelerating voltage, with polishing steps performed at 5 kV and 2 kV to achieve electron transparency. TEM and STEM imaging and EDX mapping were performed on an FEI Talos F200A.

## Results and Discussion

### Operando Optical Reaction Screening

**Figure 1:**
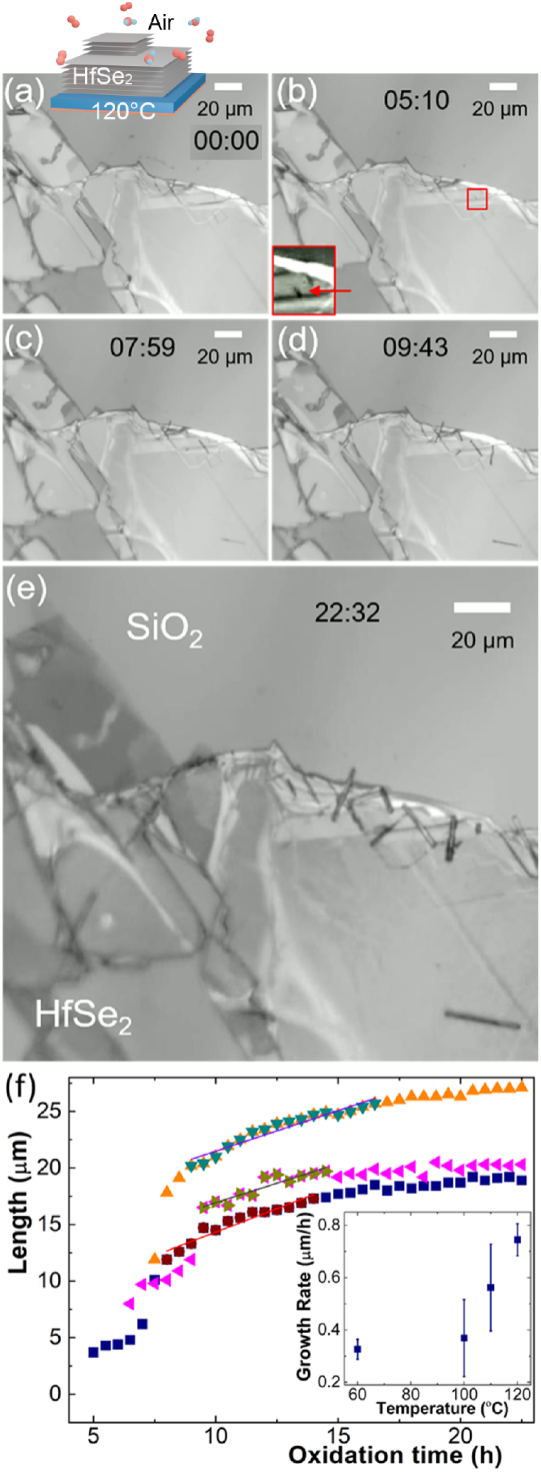
(a)–(e) Operando optical image sequence of atmospheric oxidation of a HfSe_2_ flake on a Si/SiO_2_ substrate at 120 ^◦^C. The sample setup is schematically outlined in (a). Time is indicated in the hh:mm format. The inset in (b) represents a zoom-in of the area indicated by the red square and highlights the onset of NW growth. Contrast variation that primarily reflects the formation of a Hf oxide top layer with the increasing thickness, and the corresponding depletion of the underlying HfSe_2_ is most clearly visible in the thinner HfSe_2_ regions, such as the top-left area of the flake. (f) Extrapolated length vs oxidation time growth behavior at 120 °C of individual NWs (marked by different symbols and colors; data from SI Video S1). An average corresponding growth rate was extrapolated via a linear fit as indicated. The inset plots the variation of as-extrapolated NW growth rates for different oxidation temperatures corresponding to SI Videos S1–S7.

Figure 1a–e shows a representative sequence of spectral optical images of an exfoliated HfSe_2_ flake on a Si/SiO_2_ support during atmospheric oxidation at 120 °C (see the sample schematic in Figure 1a) over a 22 h period (see Video S1). 285 nm thick SiO_2_ was chosen for enhanced optical contrast, in line with previous literature on interference contrast and optical mapping of 2D layers.^37−41^ The stack effectively forms a Fabry–Perot cavity (see Figure S1), and filtering at 730 nm most effectively reveals process signatures across the entirety of parameter space and reaction evolution (see Experimental Section and Videos S1–S3). The HfSe_2_ flakes are heterogeneous, with the initial optical contrast reflecting different terraces of varying thickness ranging from 100 to 500 nm [Figure 1a; Videos S1–S3]. Operando oxidation reveals two main features: (1) an areal time-dependent variation in optical contrast that reflects HfSe_2_ crystal oxidation and delineates the different thickness areas and (2) the localized appearance of highly anisotropic NW features.

Feature (2) can be first observed after approximately 5 h of thermal annealing, Figure 1b. After a further ∼3 h, multiple NWs become visible (Figure 1c), with a higher NW nucleation density along the HfSe_2_ flake edges and in proximity to apparent defects. The NWs grow in length, thereby leaving the focal plane or pivoting, particularly when extending beyond the boundaries of the underlying HfSe_2_ flake. This indicates that the NWs are growing on the surface of the stack. We explored the temperature dependency of the process between 30 °C and 150 °C (see Videos S1–S7). We consistently observe the aforementioned process features, albeit at 30 °C significantly longer time scales are required, and NW features are not optically discernible. No clear temperature dependence on NW nucleation times or densities could be observed likely due to the heterogeneity of the exfoliated HfSe_2_ flakes and the variability of defect sites. Figure 1f plots extrapolated NW length versus oxidation time for select individual NWs that could be optically traced for Video S1. The NW growth behavior is nonlinear, i.e., the longitudinal NW growth rates are a function of oxidation time, being highest straight after NW nucleation and then slowing down significantly. Once a NW stopped growing, it remained unchanged for the remaining oxidation time. This holds for probed oxidation at temperatures up to 150 °C (21.5 h) and times of up to 120 h (100 °C). We extrapolate an average NW growth rate via a linear curve fitting, as indicated in Figure 1f. This allows us to compare the order of magnitude of longitudinal NW growth rates for different oxidation temperatures (inset, Figure 1f). The as-extrapolated NW growth rates are seen to vary from approximately 0.3 to 0.8 μm/h for oxidation temperatures of 60 °C and 120 °C, respectively. Similar process evolution was observed without continuous focused light exposure during oxidation, indicating that the reaction does not rely on photoexcitation under these conditions. At temperatures above 220 °C, only feature (1) is observed, with no NW formation. This suggests a thermal stability window for t-Se NW growth, which coincides with the melting range of t-Se.^22,42^

### Postprocess Characterization

**Figure 2:**
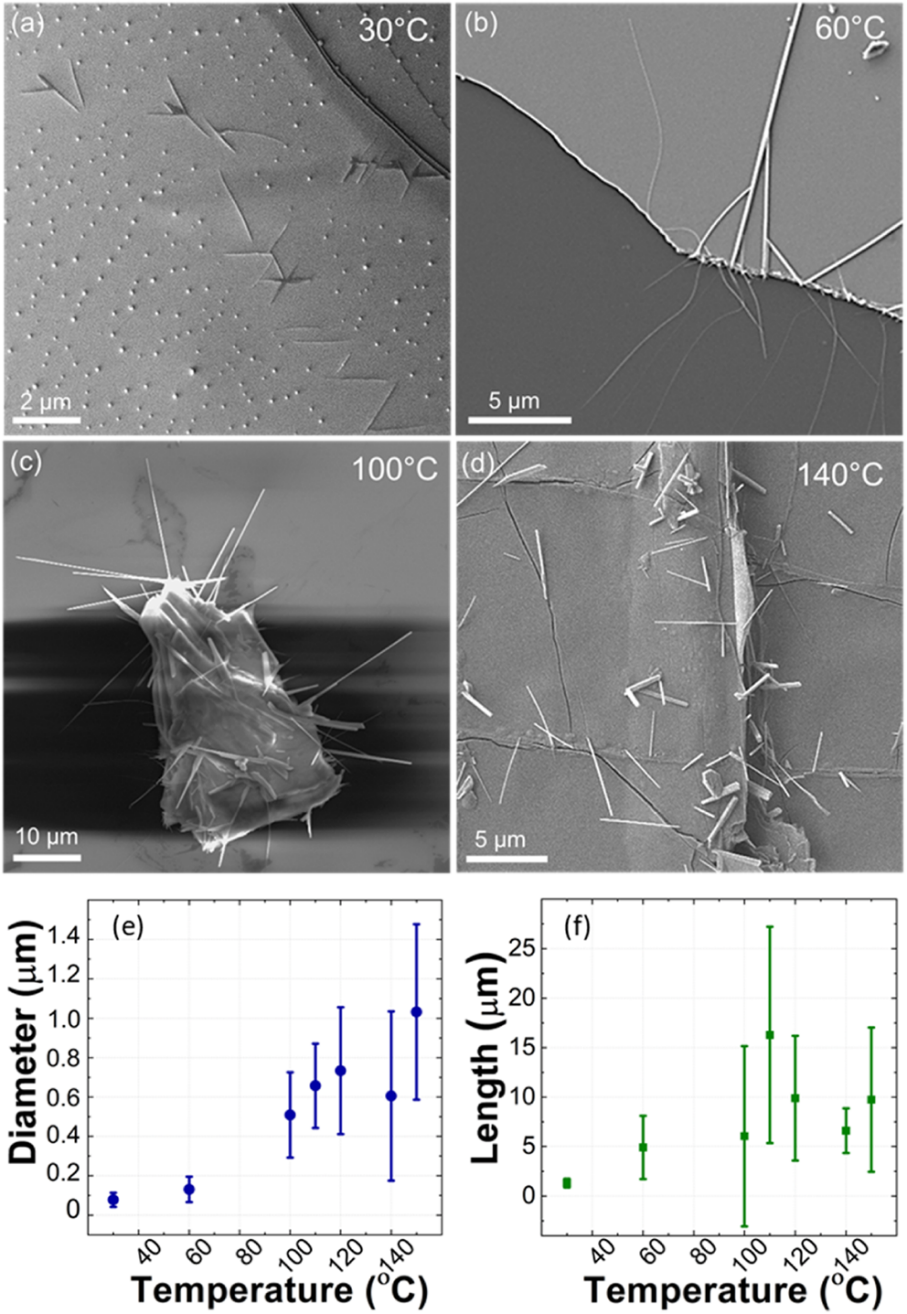
SEM analysis of SeNWs formed by atmospheric HfSe_2_ oxidation at (a) 30 °C (reaction time: 40 h), (b) 60 °C (63 h), (c) 100 °C (120 h), and (d) 140 °C (22 h 45 min). (e) Extrapolated variation of SeNW diameter and (f) SeNW length with oxidation temperature. The error bar represents the standard deviation.

Figure 2 represents postannealing SEM analysis of NW features for HfSe_2_ flakes oxidized at various temperatures and times. At 30 °C (Figure 2a), NW features are 80 nm in diameter and 1.3 μm long on average, hence the difficulty in resolving them optically. The HfSe_2_ flake surface remains relatively flat after partial oxidation, which aids feature detection and reveals a high density of small protrusions. These protrusions resemble features reported in previous studies on the ambient air stability of HfSe_2_, where they were identified as a-Se.^16,43^ The NW nucleation density is very low compared to the areal density of small protrusions. Figure 2a shows a lower areal density of protrusions for a radius of ∼1 μm around the vicinity of NW growth. In comparison, for the 60 °C sample (Figure 2b), very few such small protrusions are easily discernible. One reason for this could be the increased Se diffusivity and related tendency to agglomerate at select heterogeneous sites. We find a significantly enhanced NW nucleation density along the HfSe_2_ flake edge, particularly at edge regions that appear rough and/or decorated. Figure 2b shows NWs that have grown away from the flake onto the SiO_2_ support as well as NWs that have grown on the flake surface. For the latter case, the variation in NW diameter is much more pronounced. At higher oxidation temperatures (Figure 2c,d), larger blisters are clearly visible, and the flake surface shows cracking. There is no uniform nucleation density; rather, NW nucleation appears preferential at flake edges and cracks, specifically at higher temperatures (Figure 2d). We never observed any particle or other features attached to the growing end of a NW. While some NWs are tapered (see Figure S2), the majority are uniform in diameter. Figure 2e,f summarizes the temperature dependence of the mean and standard deviation of NW diameters and lengths extrapolated from SEM analysis. Between 30 °C and 150 °C, the mean NW diameter increases from 80 nm to 1 μm, while the standard deviation also significantly increases. In contrast, the mean NW length and its standard deviation are maximum at 110 °C. The NW aspect ratio, i.e., the ratio of length over diameter of individual NWs, reaches a maximum mean of 45 at 60 °C, where it also shows the largest standard deviation (Figure S3). These postprocess NW statistics comprise different oxidation times and should be seen in the context of the above revealed growth kinetics. Using a heater stage inside the SEM, we observe that NWs disintegrate at temperatures above ∼220 °C under vacuum conditions (Figure S4). This is consistent with the optically observed stability window and highlights that this is not linked to the presence of oxygen. Figures S5 and S6 show SEM analysis of long-term (8 months) ambient air exposures of samples originally oxidized at 60 °C and 150 °C, respectively. There is no change to as-grown SeNWs. The surface of oxidized HfSe_2_ flakes, however, continues to react and change, which manifests itself in the increased presence of small surface protrusions and roughness. For the 60 °C sample, we observe also increased agglomeration of surface features at flake/facet edges, while at 150 °C, we observe surface cracks to increasingly fill with small aggregates. This indicates that none of the oxidation conditions creates a passivation layer to sufficiently protect HfSe_2_ from continuous degradation.

**Figure 3:**
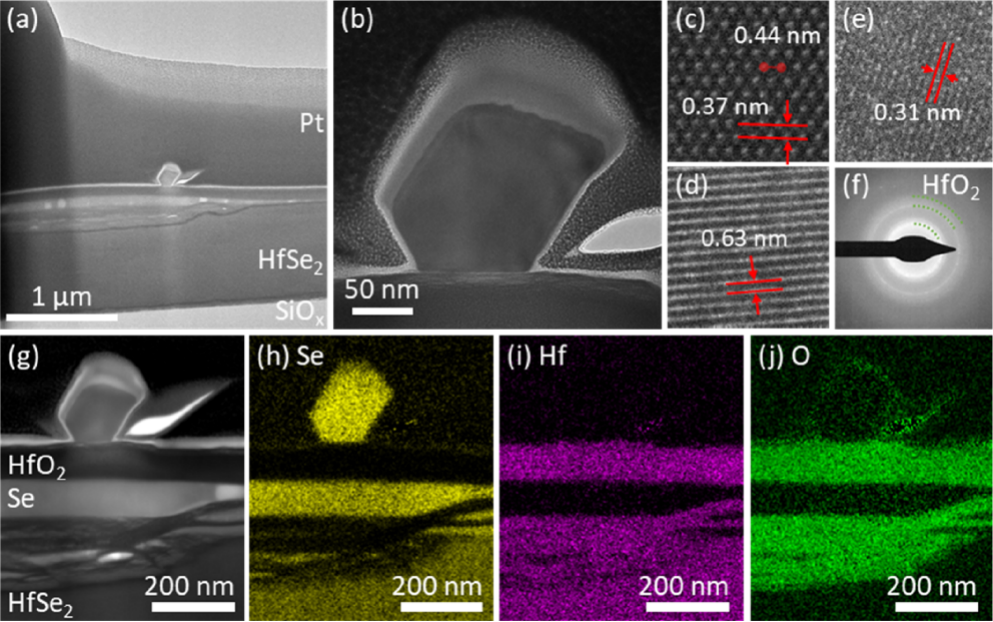
Cross-sectional TEM analysis of SeNWs formed by atmospheric HfSe_2_ oxidation at 140 °C. (a) Low magnification TEM image of the FIB lamella, with (b) showing a higher resolution image of SeNWs. (c) High-magnification image of the Se nanowire showing 0.44 nm spacing matching the [001] direction of trigonal Se. (d) High-magnification image of the HfSe_2_ flake, showing the layer distance. (e) High-magnification image of the topmost HfO_2_ layer, showing a 0.31 nm layer spacing matching the [−111] plane. (f) SAED of the HfO_2_ layer, showing that the material is mostly amorphous. (g) Bright-field STEM image of SeNWs on top of the HfO_2_ layer and corresponding (h) Se, (i) Hf, and (j) O EDS maps of the same sample region. Between HfO_2_ and HfSe_2_ are layers of HfO_*x*_ and Se. The combined thickness of the Hf oxide and a-Se layers varies between 85 and 130 nm along the cross-sectional length of 685 nm examined by the TEM lamella.

Figure 3 shows cross-sectional TEM analysis of features (1) and (2) for a sample oxidized at 140 °C. The NW is found on top of the layer stack. The higher resolution cross-sections (Figure 3b,c) reveal that the NW is fully crystalline. The lattice spacings in Figure 3c show t-Se in the [001] direction, consistent with the NWs forming via axially aligned t-Se helical chains. These findings are also consistent with further TEM analysis of NWs that were scraped off the substrate and imaged perpendicular to the growth direction (Figure S7).

Cross-sectional analysis confirms that the initial HfSe_2_ flake primarily oxidized from the top with a thinner layer of oxidation at the SiO_*x*_ interface (see Figure S8). The buried crystalline, layered 1T HfSe_2_ remained intact (Figure 3d). The topmost layer consists of nanocrystalline HfO_2_, with selected area diffraction (SAED) patterns indicating a monoclinic HfO_2_ phase (Figure 3e,f). There is no well-defined interface or interaction between this top layer and the SeNW (see Figure S9). Figure 3g–j provides a cross-sectional STEM image and relevant energy dispersive X-ray spectroscopy (EDS) elemental maps that confirm the presence of Se in two regions: a pure Se layer underneath the nanocrystalline HfO_2_ layer and Se interspersed within the HfO_*x*_ layers above the HfSe_2_ flake. SAED reveals this buried Se layer to be amorphous with a thickness that varies from 50 nm down to 5 nm, being present across the entire cross-section. EDS mapping further reveals that amorphous Se is segregated from the Hf oxide, with no indication of mixed oxy-selenide phases, in contrast to what is typically observed for the oxidation of HfS_2_, where oxy-sulfide phases are commonly reported.^11,44^ The accumulation of pockets of Se—within, below, or on top of the Hf oxide—creates increased surface roughness and blister formation.

In samples oxidized at 30 °C, cross-sectional TEM reveals a layered HfO_*x*_/Se mixed region on the surface but not within the HfSe_2_ bulk (Figure S10). The surface structure is consistent with nanocrystalline sheets of HfO_*x*_ sandwiching a-Se aggregates, i.e., a similar morphology as for the 140 °C sample, but with thinner oxide layers and lacking the HfO_2_ layer. The NW found on the stack surface shows crystallinity (Figure S10c), likely with the same t-Se structure as the higher-temperature sample.

**Figure 4:**
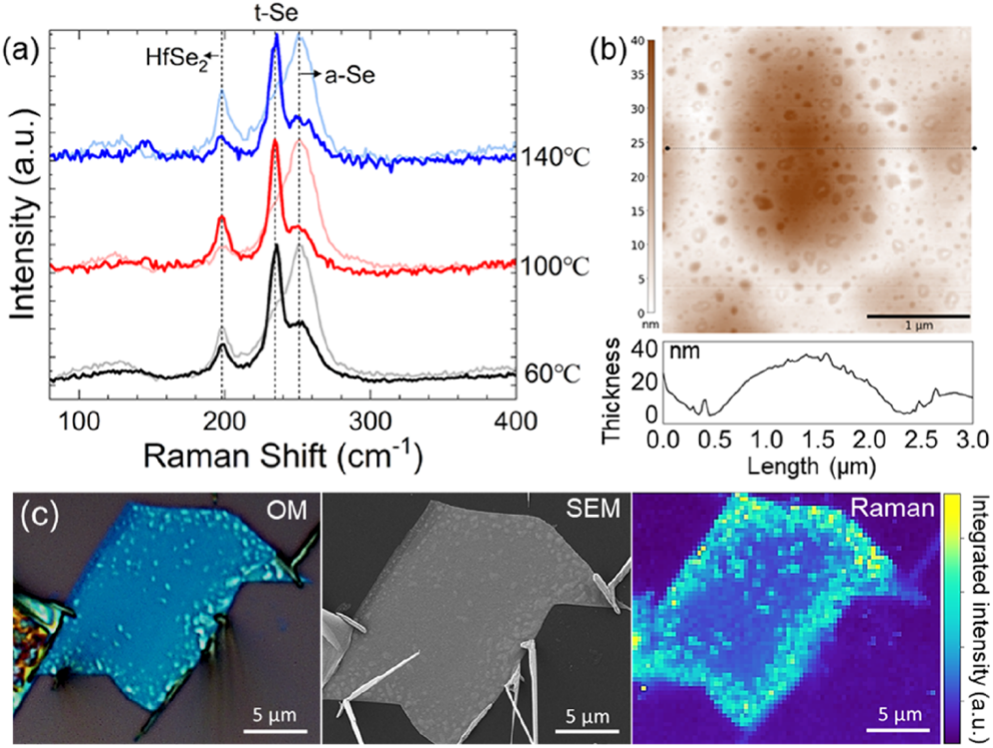
(a) Raman spectra after atmospheric HfSe_2_ oxidation at various temperatures. The lighter and solid lines represent measurements of areas without and with a NW present, respectively. Raman analysis at 30 °C was omitted as the NWs could not be optically located. (b) AFM surface analysis of the 140 °C sample, with the line scan profile from the indicated position. (c) Optical microscopy image, SEM image, and Raman map of the HfSe_2_ flake oxidized at 100 °C for 48 h. The Raman mapping was performed at 532 nm excitation, with a power density of approximately 0.06 mW cm^–2^ and with a step size of 0.3 μm and 1 s measurement time per pixel. The intensity reflects the integrated area under the spectral range of 220–270 cm^–1^ computed using Simpson’s rule, providing a quantitative measure for the distribution of Se across the sample region. Not all SeNWs are shown in the Raman map as they were out of the focal plane.

Figure 4a shows Raman analysis at 514 nm excitation of HfSe_2_ flakes oxidized at 60 °C, 100 °C, and 140 °C. For each sample, we compare a region with and without SeNWs, both measured on top of the stack. In the absence of NWs, the Raman spectra for all samples show a peak centered around 199 cm^–1^ and peaks centered around 234 cm^–1^ and 250 cm^–1^. The first can be consistently assigned to the out-of-plane A_1_g optical phonon mode of 1T HfSe_2_.^45^ The peaks at 234 cm^–1^ and 250 cm^–1^ can be assigned to characteristic modes of amorphous-like Se.^46−48^ The peak around 234 cm^–1^ is thereby typically attributed to a bond-stretching vibrational mode of chains in t-Se-like configuration, whereas the peak around 250 cm^–1^ is attributed to a bond-stretching vibrational mode of disordered Se chains. We observe the 250 cm^–1^ peak to be typically dominant in the absence of SeNWs. The literature has reported a complete shift from the 250 cm^–1^to the 234 cm^–1^ peak due to structural conversion upon irradiation with higher laser power and above band gap photon energy or upon heating.^48^ We do not observe significant spectral changes while measuring at comparable laser powers (0.06–1 mW cm^–2^) with 514, 532, or 633 nm excitation. We do, however, observe lateral and thickness-dependent (i.e., changes with laser focus) variation in the relative intensities between these two peaks, without any clear correlation to oxidation temperature. For sample spots that include SeNWs, we observe for all temperatures a dominant peak centered around 234 cm^–1^. Raman spectra of individual SeNWs that have grown onto the SiO_2_ substrate, i.e., without the background of an underlying flake (Figure S11), are consistent with previously reported SeNW literature,^28,49^ showing a dominant 234 cm^–1^ peak, which matches the reported A_1_ mode of t-Se, and minor peaks at 439–460 cm^–1^ and 147 cm^–1^, being attributed to second-order spectra of t-Se and a transverse optical phonon mode (E mode), respectively. At high (0.6 mW cm^–2^) laser power densities, we observe SeNWs to get damaged, which based on SEM analysis, we attribute to excessive local heating (see Figure S12).

Figure 4b shows AFM analysis of the surface of a HfSe_2_ flake after oxidation at 140 °C. We observe blisters of ∼2 μm lateral extent and ∼30 nm height. This is consistent with our TEM analysis, Figure 3a. In between these blisters, smaller protrusions can be seen on the surface, typically less than ∼10 nm in height. This is consistent with our SEM analysis in Figure 2, with AFM being able to resolve these small protrusion also in the presence of higher surface roughness for 140 °C oxidation. Figure 4c shows correlative mapping of a HfSe_2_ flake oxidized at 100 °C by optical microscopy, SEM, and Raman. For the latter, the mapped intensity reflects the integrated area under the spectral range of 220–270 cm^–1^, which covers all Se-related peaks and thus highlights the distribution of Se across the sample region. Consistent with our TEM analysis, we find enhanced Raman intensity matched to blisters (see also Figure S13 where correlative sample mapping is more focused on such blisters). We also find Se enrichment around the flake edges, again matched to blisters and roughness seen optically and by SEM. As the SeNWs are seen to originate from these areas, this supports the argument that the observed SeNW growth is fed from Se created by the oxidation reaction.

### Discussion of Underpinning Mechanisms

**Figure 5:**
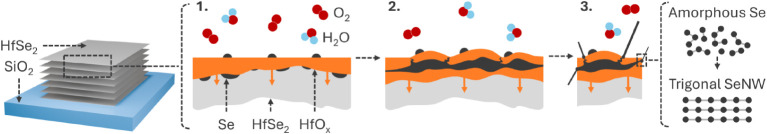
Schematic of proposed growth mechanism: (1) The oxidation of a 2D HfSe_2_ crystal produces a Hf oxide layer and Se. Oxidation progresses at the oxide–selenide interface, thus producing increasingly more buried Se. (2) This Se accumulates in blisters, leading to roughness and cracking of the surface oxide. (3) Se can diffuse through cracks or to the crystal side, and at select nucleation sites, SeNWs crystallize and grow.

Figure 5 schematically summarizes the proposed solid-state reaction scheme, with the oxidation of a 2D selenide locally producing Se that then feeds SeNW growth. This coupling brings together hitherto unconnected literature on the oxidation of TMDs and the related reaction anisotropies for such 2D materials, the crystallization of Se, and growth of 1D SeNWs. HfSe_2_ oxidation in this context refers to the formation of an Hf oxide, i.e., the metal constituent of the TMD. A crucial aspect, which has received less attention in the TMD literature is what happens to the chalcogen constituent. The oxidation of metallic Hf has been shown to proceed by the inward diffusion of oxygen as ions through anion vacancies in hafnia, i.e., the oxide grows at the buried interface.^50,51^ Se will hence form at the hafnia–HfSe_2_ interface. The nature of Se segregation will depend on Se diffusivity within the hafnia and HfSe_2_, as well as the relation of this diffusivity to the rate of oxidation, i.e., the time-dependent rate at which new Se and hafnia will be produced. We find that for all temperatures (30–150 °C), Se aggregates and forms blisters within the hafnia and extended interfacial films at the hafnia–HfSe_2_ interface. Where the hafnia–HfSe_2_ interface is sufficiently buried, as is found at the higher temperatures (60–150 °C), the Se diffusivity through the hafnia is not sufficient for it to reach the top surface, and the Se vapor pressure^22^ is not sufficiently high to volatize all the Se that reaches the surface. While the Se at the surface forms clusters, trapped Se progressively deteriorates the hafnia film, leading to increased porosity, roughness, and cracks, which in turn affects the Se transport as oxidation continues.

The emergence of t-Se NWs requires nucleation. We note that previous literature reports the crystallization of a-Se films within the temperature range of 50–200 °C and well within the time scale of our experiments.^32,52^ Such film crystallization studies do not report NW formation. It is known that Se crystallization kinetics can be dependent on the detailed structure of a-Se linked to the thermal and exposure history of the material.^25,26^ A key difference for our scenario is that the 1D SeNW nucleation and growth kinetics are closely coupled to the oxidation kinetics of 2D HfSe_2_. A relatively high rate of buried Se creation can kinetically lead to a lack of crystallization. Our operando data show that SeNW growth occurs in isothermal conditions, i.e., we can exclude quenching as a necessity for such crystal nucleation.^28,49^ Most literature reports t-Se NW growth to emerge from a-Se particles with dimensions much larger than the NW diameter.^28,49,53^ Se chains are thought to evolve into t-Se seeds via preferential Se addition to the chain ends, thus promoting anisotropic growth along the *c*-axis.^22,25^ To date, very little is known about the actual reaction kinetics that lead to SeNW growth. Our data clearly shows that the nucleation site can be different from the site of the Se source. While the Se is created at a buried interface, we observe t-Se NW formation only at the stack surface. For NW nucleation to occur, a Se diffusion feed is required to allow Se to reach the surface, hence the observed proximity to cracks and flake edges. The film structure and surface of the created hafnia significantly vary between 30 and 150 °C, yet crystalline SeNW formation can be observed for all these conditions. The NW growth here is not epitaxial nor templated. The low NW nucleation yield and site selectiveness indicate that heterogeneous nucleation sites can play an important role. Various Se precursor reactions can be activated by metal nanoparticles, such as Au and Ag, including dislocations,^54^ leading to heterogeneous nucleation of Se. We are not aware of any reports of site-selective Se crystallization, but based on our data, it can be speculated that specific, localized hafnia (defect) sites activate t-Se NW nucleation. Even for extended times, we find only 1D-shaped NW crystals, and no Se nanosheets or belts have been reported for other methods and are thought to follow vapor–solid (VS) growth mechanisms^53^ or are seeded via selective surfaces.^55^ We find the mean NW diameter to positively correlate with oxidation temperature. We also find that NWs on the stack surface are more likely to develop larger diameters than NWs that grow away from the flake. This indicates that depending on the Se diffusion rate, the NW diameter can further increase after nucleation. The reaction anisotropy between the Se chain end and side walls is so large that typically the diameter increases for the entire length of the NW. Thus, only rarely is a tapered NW formed. Our operando data finds time-dependent longitudinal NW growth rates. This can be seen to link to the diffusive Se feeding. We refrain, however, here to plot extrapolated longitudinal NW growth rates in Arrhenius fashion, mainly due to the complex coupling of Se diffusion to its creation via the oxidation reaction, both of which are thermally activated. The observed stopping of longitudinal NW growth points to a loss of Se supply. These coupled growth kinetics lead to the observed statistical distributions of NW dimensions.

## Conclusions

We demonstrated that the oxidation of a 2D TMD crystal can serve as a local chalcogen source, which can be utilized as a precursor to localized structural formation and nanowire crystal growth. The approach is versatile, with the material source being simply an exfoliated flake. The localized, surface-bound process opens pathways to operando reaction characterization. We focused on HfSe_2_, motivated by the model material character of Se and hitherto disconnected literature on TMD oxidation, Se film polymerization and crystallization, and synthetic SeNW growth approaches. Our results underscore the need of taking into account the chalcogen constituent when studying TMD oxidation, in line with previous studies on ambient TMD stability. Compared to the many previous chemistry and confined SeNW growth approaches, our solid-state approach involves no solvents and is template-free. This allows for the minimization of possible contamination and impurities. It also endows compatibility with integrated process technology as used in the semiconductor industry. We find that crystallization into t-Se NWs only occurs at the stack surface or edges, i.e., that the Se source is separated from the site of NW nucleation. This motivates new process design avenues and future studies on achieving nucleation control and higher yield via, e.g., defect and/or impurity seeding. An application consideration for the latter is that it can degrade product purity, an aspect widely discussed in the context of metal-seeded vapor–liquid–solid (VLS) NW growth. We find individual SeNWs to emerge from small (<1 μm) HfSe_2_ crystals (see Figure S12), which highlights the potential to increase reaction localization. Recent data mining identified 487 materials that consist of weakly bonded 1D molecular chains,^56^ which can serve as building blocks to ultrascaled and more energy-efficient device designs. Our platform opens opportunities for the high-throughput experimental process discovery for such low- and mixed-dimensional integrated device material portfolios. Our work motivates such process discovery to be holistic, i.e., to transcend the current isolated material focus, particularly to explore in situ, in-place synthesis and synergistic reaction designs to drive scalable heterogeneous integration technologies, thus unlocking technological potential in key application areas, such as quantum electronics, energy-efficient information, communication and data storage technologies, and biomedical devices.

## Supporting Information Available

Process details and additional optical, Raman, SEM, and TEM characterization data (PDF)
Optical microscopy of HfSe_2_ flake oxidised at 120 °C in ambient for 22 h 40 min (captured wavelength = 730 nm) (MP4)
Optical microscopy of HfSe_2_ flake oxidised at 120 °C in ambient for 22 h 40 min (captured wavelength = 590 nm) (MP4)
Optical microscopy of HfSe_2_ flake oxidised at 120 °C in ambient for 22 h 40 min (captured wavelength = 450 nm) (MP4)
Optical microscopy of HfSe_2_ flake oxidised at 110 °C in ambient for 21 h 20 min (captured wavelength = 730 nm) (MP4)
Optical microscopy of HfSe_2_ flake oxidised at 100 °C in ambient for 120 h (captured wavelength = 570 nm) (MP4)
Optical microscopy of HfSe_2_ flake oxidised at 100 °C in ambient for 23 h (captured wavelength = 730 nm) (MP4)
Optical microscopy of HfSe_2_ flake oxidised at 60 °C in ambient for 40 h (captured wavelength = 730 nm) (MP4)
