# Supplementary material for: Se Nanowire Crystal Formation via Oxidation of 2D HfSe2: A Solid-State, In Situ Reaction Coupling for Heterogeneous Integration Technologies
Source: ACS Appl Nano Mater. 2025 Apr 9;8(15):7608–15. doi: 10.1021/acsanm.5c00308 (PMC12012781; doi:10.1021/acsanm.5c00308)
Supplement: Supplementary file 1 — an5c00308_si_001.pdf [file an5c00308_si_001.pdf]

Supporting Information: Se Nanowire Crystal Formation via Oxidation of 2D $HfSe_2$: A Solid-State, In Situ Reaction Coupling for Heterogeneous Integration Technologies

Sunvir Sahota,[†] Irina Chircă,[†] Oliver J. Burton,[†] Hao Yu,[†] Max Rimmer,[‡] Jinfeng Yang,[†] Kyungseo Park,[†] Arthur Summers,[†] Siddika Mertdinc-Ulkuseven,[†,¶] Matthew Lindley,[‡] Sarah J. Haigh,[‡] and Stephan Hofmann[*,†]

†*Department of Engineering, University of Cambridge, Cambridge CB3 0FA, UK*

‡*Department of Materials, University of Manchester, Oxford Road, Manchester M13 9PL, UK*

¶*Metallurgical and Materials Engineering Department, Istanbul Technical University, 34469, Maslak, Istanbul, Turkey*

E-mail: sh315@cam.ac.uk

Video S1: Optical microscopy video of $HfSe_2$ flake being oxidised at 120 °C in ambient for 22 h 40 min. This was captured at a wavelength of 730 nm, and is the video counterpart of Figure 1.

Video S2: Optical microscopy video of $HfSe_2$ flake being oxidised at 120 °C in ambient for 22 h 40 min. Difference to video S1 is that this was captured at a wavelength of 590 nm.

The difference in intensities reflects the results shown in SI Figure S1. Over our time period of oxidation it can be seen that the contrast most evidently changes at 730 nm.

Video S3: Optical microscopy video of $HfSe_2$ flake being oxidised at 120 °C in ambient for 22 h 40 min. This video was captured at a wavelength of 450 nm.

Video S4: Optical microscopy video of $HfSe_2$ flake being oxidised at 110°C in ambient for 21 h 20 min. This was captured at a wavelength of 730 nm. This video particularly highlights the stochastic nature of NW nucleation with some flakes producing NWs but others not (within the resolution of the microscope).

Video S5: Optical microscopy video of $HfSe_2$ flake being oxidised at 100 °C in ambient for 120 h. This was captured at a wavelength of 570 nm. Due to the long time scale the Fabry Perot cycles can be seen as portions of the flake go from white to black to white again. Secondly, the NWs at longer time scales appear to have a degree of freedom in their movement as they can be seen to go in and out of the focal plane. Finally, at long time scales the NWs do not appear to continuously keep growing in length, even though the flake oxidation is still progressing.

Video S6: Optical microscopy video of $HfSe_2$ flake being oxidised at 100 °C in ambient for 23 h. This was captured at a wavelength of 730 nm. The video highlights the stochastic nature of time of NW nucleation, as this is heated at the same temperatures as video S5,where NWs are first observed around 5 h, however here they form after over 10 h.

Video S7: Optical microscopy video of $HfSe_2$ flake being oxidised at 60 °C in ambient for 40 h. This was captured at a wavelength of 730 nm.

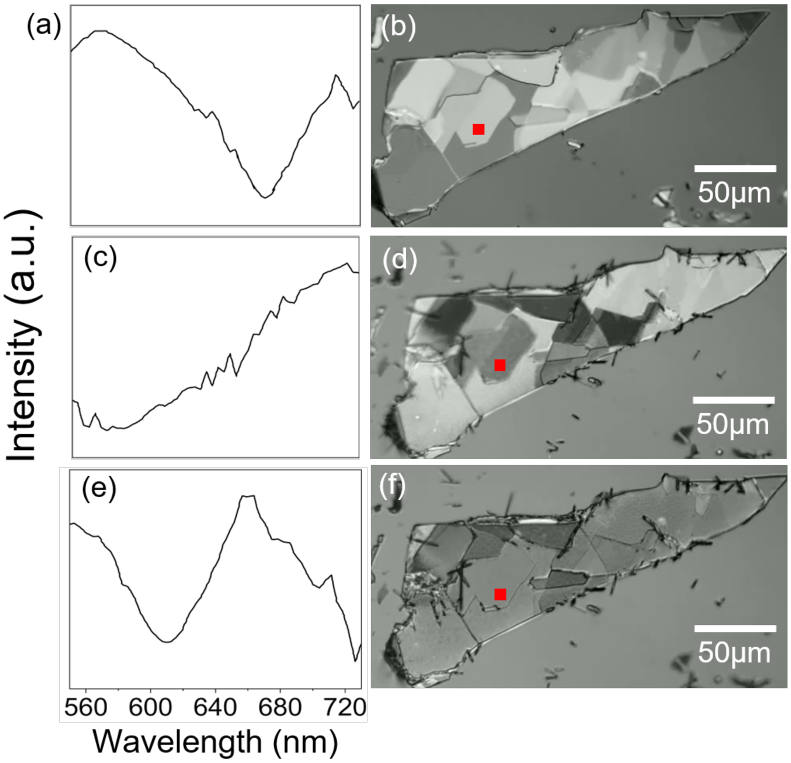

Figure S1: Feature (1) contrast variation for the oxidation of $HfSe_2$ flake at 100°C. Plots (a), (c) and (e) correspond to the intensity at the square marked in red (actual corresponding spot size 0.24 μm^2) in the respective optical images (b), (d) and (f). From these plots it can be seen that the wavelength of the maximum intensity changes at different time steps of the oxidation, due to the changing optical path length of the stack. The stack acts as a Fabry Perot resonator, whose spectral response depends on the cavity thickness and refractive index, which change with oxidation.

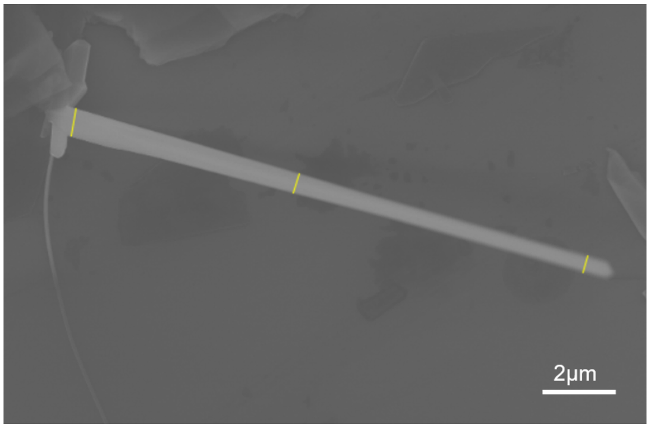

Figure S2: SEM image showing a tapered SeNWs emerging from a $HfSe_2$ flake oxidised at 100°C for 120 h in air. From left to right the lengths of the yellow lines are 0.77, 0.55 and 0.46 μm, respectively.

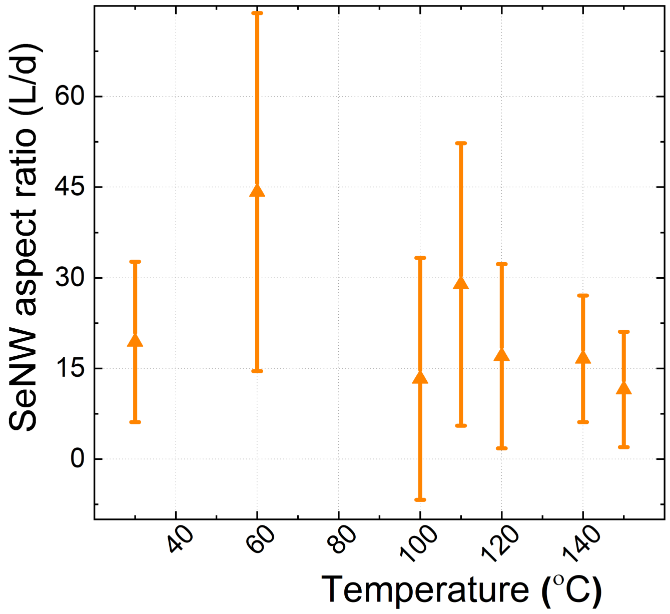

Figure S3: Extrapolated temperature variation of SeNW aspect ratio, i.e. ratio of length over diameter, based on SEM analysis. The error bar represents standard deviation.

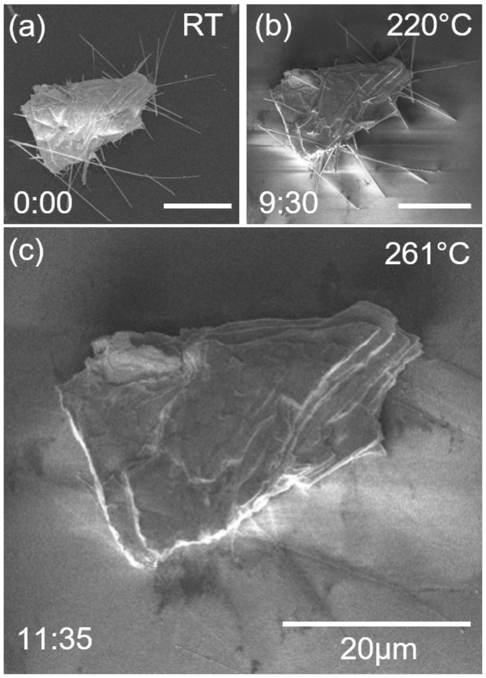

Figure S4: In situ heating in vacuum of t-Se NWs inside the SEM. The temperature was linearly ramped at 20 °C/min using a Kammrath and Weiss 1050 heating module. NWs were initially produced via oxidation at 100 °C for 120 h. (a)-(c) reflect time sequence (time in mm:ss) as well as the temperature ramp. We observe SeNW degradation at temperatures above the melting point of t-Se. Scale bar 20 μm for all images.

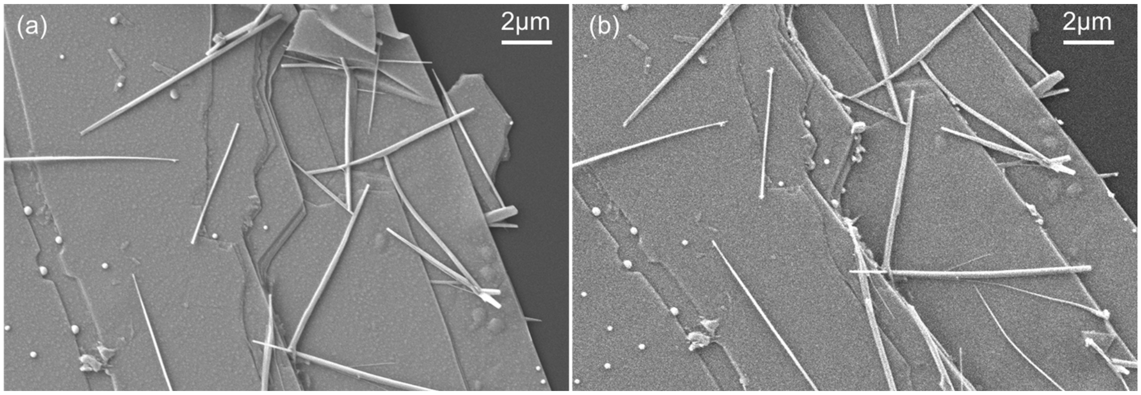

Figure S5: Effect of long term air exposure on $HfSe_2$ sample oxidised at 60 °C: SEM images of representative sample area (a) after one month and (b) after 8 months of ambient air exposure.

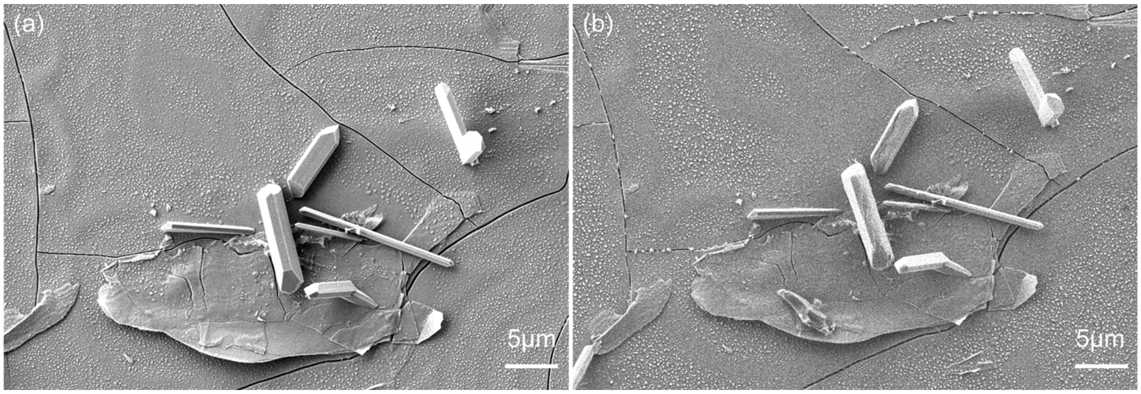

Figure S6: Effect of long term air exposure on $HfSe_2$ sample oxidised at 150 °C: SEM images of representative sample area (a) after 18 days and (b) after 8 months of ambient air exposure.

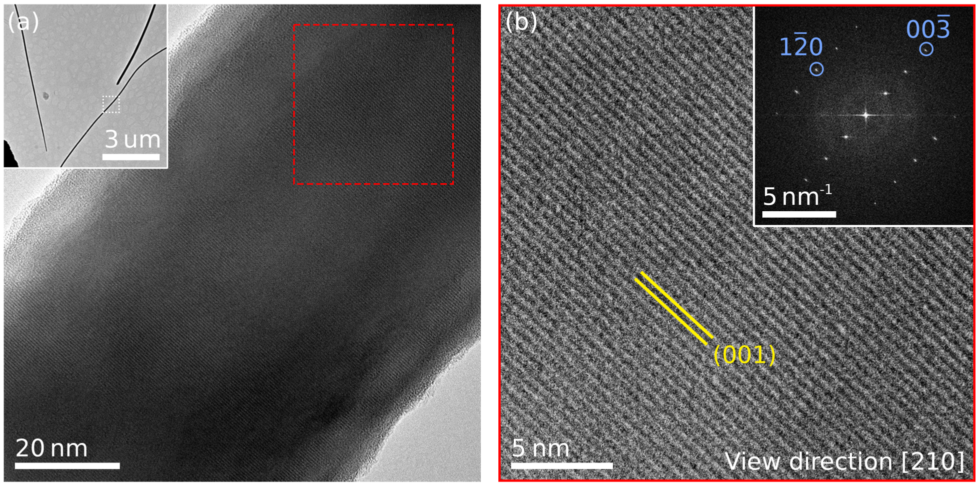

Figure S7: TEM analysis of NWs scraped off a $HfSe_2$ flake onto a lacey carbon TEM grid and imaged in the [100] direction. The NWs were grown via oxidisation at 100°C for 65 h in ambient without optical microscope illumination. The white square within inset of (a) indicates the position of the NW segment shown at higher resolution. (b) Higher resolution of region indicated as red square within (a), imaged along the [210] zone axis, with FFT analysis of (b) inset. A lattice spacing of 0.49 nm for (001) planes matches that of the t-Se structure. The decrease in image contrast seen at the NW edges in (a) is due to the beam-induced amorphization of the Se during imaging.

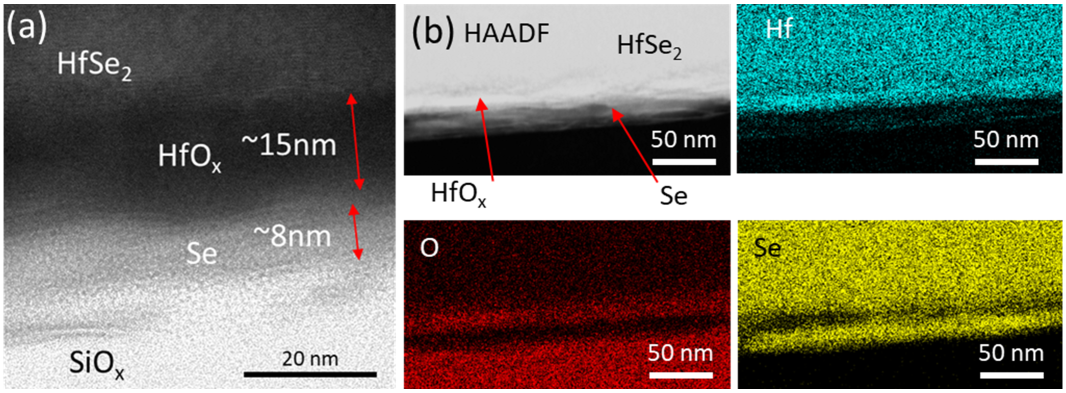

Figure S8: (a) TEM of the bottom interface of the $HfSe_2$ sample after 140 °C oxidation. An oxide layer has formed with thickness of ≈15 nm, with ≈8 nm of Se between the substrate and the flake. This is significantly thinner than at the top surface, and no nanocrystalline HfO_2 layer is present. (b) STEM-EDX showing the distribution of Hf, O and Se in the same region. The data shows that a HfO_x layer has formed at the bottom of the $HfSe_2$ flake, with a layer of Se between the sample and the SiO_2 substrate.

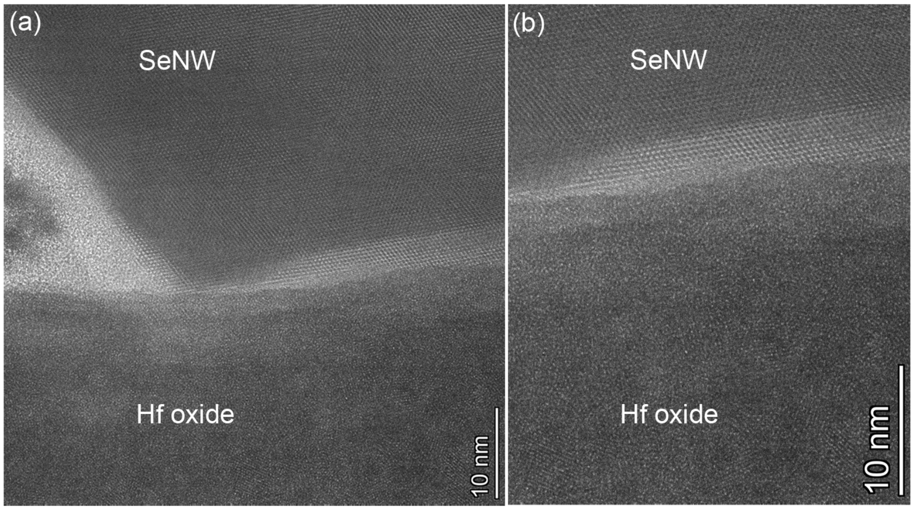

Figure S9: (a), (b) Cross-sectional TEM images of the SeNW-Hf oxide interface of sample shown in Figure 3 ($HfSe_2$ sample after 140 °C oxidation). There is no observable well-defined/epitaxial interaction.

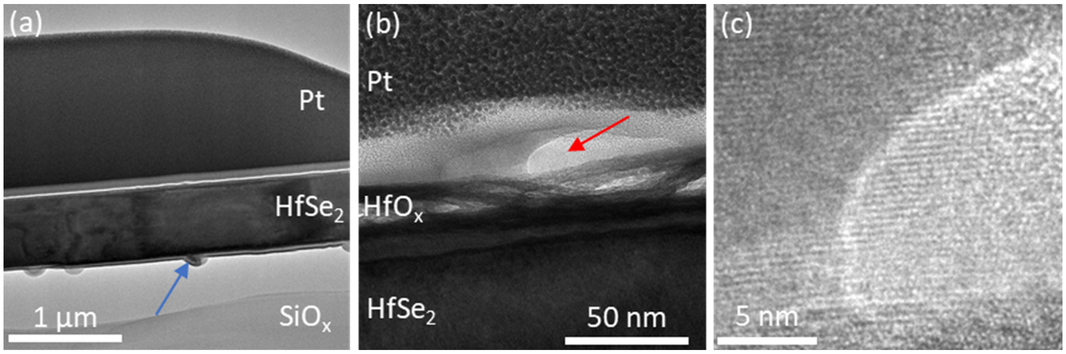

Figure S10: Cross-sectional TEM image of sample oxidised at 30 °C for 40 h in ambient conditions. Note that the $HfSe_2$ flake has delaminated from the SiO_2 substrate during milling and the object on bottom of the flake (blue arrow) in (a) is redeposited Si created during the FIB lamella preparation process. (b) TEM image of region identified as Se NW (red arrow). The top layer of HfO_x is thinner than was found for the HfO_x in the 140 °C sample and has a layered morphology with trapped bubbles between the layers. (c) Higher magnification TEM imaging reveals the interlayer spacing within the NW, which matches t-Se consistent with the NWs formed at 140 °C. Further imaging was prevented due to high rates of beam damage for this sample, rapidly degrading the NW crystallinity.

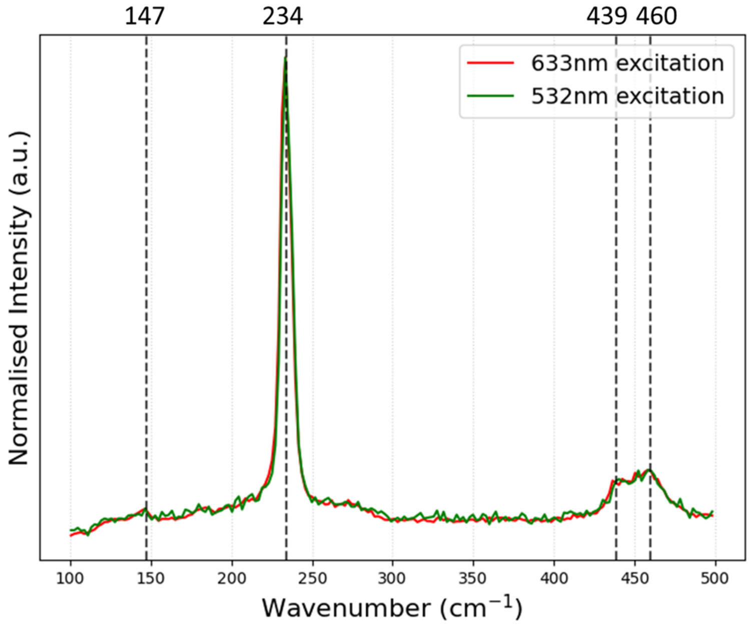

Figure S11: Raman analysis of SeNW grown onto SiO_2 substrate from $HfSe_2$ sample oxidised at 100°C (24 h).

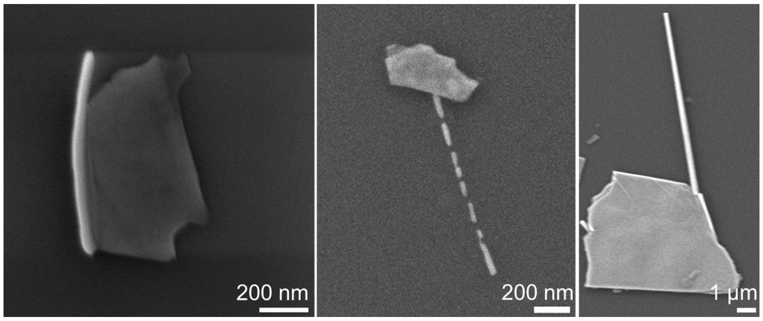

Figure S12: SEM images of SeNWs emerging from small $HfSe_2$ crystals oxidised at 100 °C. The middle image shows NW damage after Raman exposure at a high power density of approx. 0.6 mW cm^{-2} at 532 nm excitation. The damage indicates that excessive heating of the SeNW occurred at this power level.

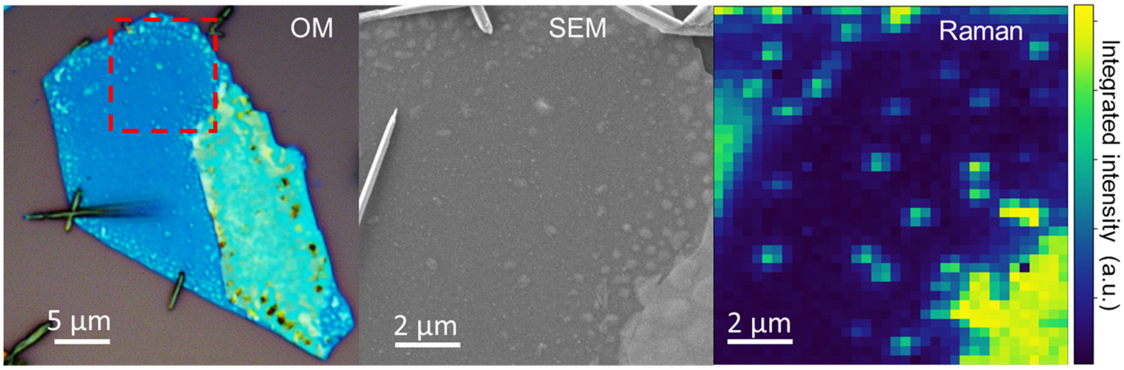

Figure S13: Optical microscopy, SEM and Raman map of same $HfSe_2$ sample area oxidised at 100 °C (48 h).
